# Cross-Attention Adaptive Feature Pyramid Network with Uncertainty Boundary Modeling for Mass Detection in Digital Breast Tomosynthesis

**DOI:** 10.3390/bioengineering12020196

**Published:** 2025-02-17

**Authors:** Xinyu Ma, Haotian Sun, Gang Yuan, Yufei Tang, Jie Liu, Shuangqing Chen, Jian Zheng

**Affiliations:** 1 School of Biomedical Engineering (Suzhou), Division of Life Sciences and Medicine, University of Science and Technology of China, Hefei 230026, China; xinyuma@mail.ustc.edu.cn (X.M.);; 2 Suzhou Institute of Biomedical Engineering and Technology, Chinese Academy of Sciences, Suzhou 215163, China; 3 Shandong Laboratory of Advanced Biomaterials and Medical Devices in Weihai, Weihai 264200, China; 4 Gusu School, Nanjing Medical University, Suzhou 215006, China; 5 Department of Radiology, The Affiliated Suzhou Hospital of Nanjing Medical University, Suzhou 215000, China

**Keywords:** digital breast tomosynthesis, computer-aided detection, mass detection, medical imaging

## Abstract

Computer-aided detection (CADe) of masses in digital breast tomosynthesis (DBT) is crucial for early breast cancer diagnosis. However, the variability in the size and morphology of breast masses and their resemblance to surrounding tissues present significant challenges. Current CNN-based CADe methods, particularly those that use Feature Pyramid Networks (FPN), often fail to integrate multi-scale information effectively and struggle to handle dense glandular tissue with high-density or iso-density mass lesions due to the unidirectional integration and progressive attenuation of features, leading to high false positive rates. Additionally, the commonly indistinct boundaries of breast masses introduce uncertainty in boundary localization, which makes traditional Dirac boundary modeling insufficient for precise boundary regression. To address these issues, we propose the CU-Net network, which efficiently fuses multi-scale features and accurately models blurred boundaries. Specifically, the CU-Net introduces the Cross-Attention Adaptive Feature Pyramid Network (CA-FPN), which enhances the effectiveness and accuracy of feature interactions through a cross-attention mechanism to capture global correlations across multi-scale feature maps. Simultaneously, the Breast Density Perceptual Module (BDPM) incorporates breast density information to weight intermediate features, thereby improving the network’s focus on dense breast regions susceptible to false positives. For blurred mass boundaries, we introduce Uncertainty Boundary Modeling (UBM) to model the positional distribution function of predicted bounding boxes for masses with uncertain boundaries. In comparative experiments on an in-house clinical DBT dataset and the BCS-DBT dataset, the proposed method achieved sensitivities of 89.68% and 72.73% at 2 false positives per DBT volume (FPs/DBT), respectively, significantly outperforming existing state-of-the-art detection methods. This method offers clinicians rapid, accurate, and objective diagnostic assistance, demonstrating substantial potential for clinical application.

## 1. Introduction

Breast cancer is a prevalent and highly impactful type of cancer affecting women, accounting for a substantial proportion of cancer-related deaths worldwide [1]. Early diagnosis is crucial for effective treatment and positive prognostic outcomes [2]. Among the early symptoms, the presence of a breast mass is of particular clinical significance. A breast mass is an abnormal localized lesion within the breast tissue, characterized by either complete or partial outward projection of the margins and increased density at the lesion center compared to the periphery. Accurate localization of these masses is essential in breast computer-aided diagnosis (CAD) systems. Clinically, digital breast tomosynthesis (DBT) serves as a primary modality for early breast cancer screening, which uses 3D slice imaging of the breast. DBT demonstrates advantages in mass detection and significantly enhances diagnostic accuracy, making it a commonly used modality for early breast cancer screening in recent years. However, DBT generates a significantly larger volume of data, resulting in a prolonged and intense interpretation workload. This not only diminishes the diagnostic accuracy of physicians but also introduces inconsistency in evaluations [3,4]. In recent years, deep learning-based CAD systems have emerged in the field of medical image diagnosis. These systems provide clinicians with reliable and objective references during the diagnostic process, effectively alleviating the complexities of DBT interpretation and enhancing diagnostic efficiency and accuracy [5,6,7].

**Figure 1:**
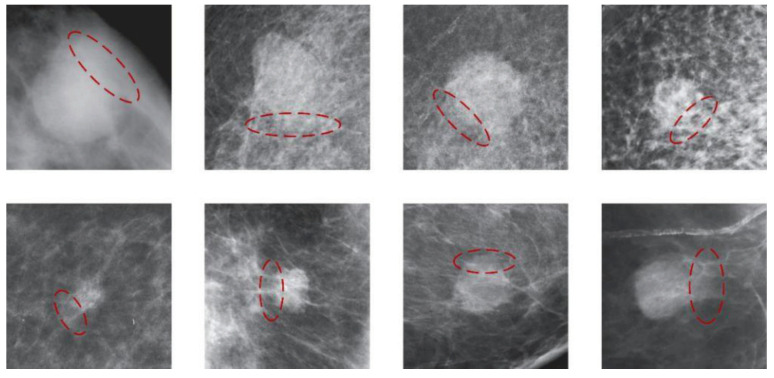
The illustration of blurred mass edges or obscured by dense glandular tissue. Blurry edges are indicated by red dashed ellipses.

Recent years have witnessed the impressive success of convolutional neural networks (CNNs) in DBT breast mass detection. However, these methods still have limitations in breast mass-detection tasks due to several reasons:

(1) Masses vary in size and appearance, posing significant challenges in effectively integrating multi-scale features, which classic CNN-based object-detection algorithms like the Feature Pyramid Network (FPN) often struggle to address. Analysis of the Breast Imaging Reporting and Data System (BI-RADS) [8] reveals that most malignant masses exhibit irregular shapes, indistinct margins, and characteristic details such as spiculations or lobulations. The pixel-wise addition approach employed by FPN for feature fusion is overly coarse, failing to effectively merge high-level semantic features with low-level detailed features. Furthermore, the top-down hierarchical feature fusion in FPN primarily emphasizes feature interaction between adjacent layers, resulting in inefficient propagation of high-level semantic features to lower layers.

(2) Breast mass lesions with high-density or iso-density characteristics exhibit minimal visual distinction from partially dense glandular tissue, rendering them susceptible to false positives in traditional CNN detection algorithms. The light radiographic appearance of the breast on mammography is a strong risk factor for breast cancer [9]. In traditional CNN algorithms, dense glandular regions within the background are given equivalent significance as other tissue types in the feature maps forwarded to the classifier, which results in a failure to effectively discriminate between mass lesions and dense glandular regions.

(3) Partial blurring or occlusion of mass boundaries leads to uncertainty in labeling. As shown in Figure 1, the ambiguity surrounding mass boundaries significantly impacts the accuracy of boundary delineation by medical professionals. Due to the indistinct mass boundaries, the model struggles to extract pertinent information near the labeled box, impeding accurate localization and thereby complicating model optimization.

To address these challenges, we propose a novel network architecture called CU-Net for breast mass detection in DBT. The primary contributions of this article are summarized as follows:

(1) A novel DBT mass-detection method is presented, demonstrating proficient and direct feature fusion across multi-scale feature maps to achieve better detection performance.

(2) We propose the Cross-Attention Adaptive Feature Pyramid Network (CA-FPN) with the Breast Density Perceptual Module (BDPM), which enhances inter-feature interaction across various scales and enhances the model’s focus on dense breast regions.

(3) We propose the Uncertainty Boundary Modeling (UBM) framework, which models the distribution function of predicted box positions, providing uncertainty estimates for the predicted coordinates and yielding more precise bounding box predictions.

## 2. Related Works

### 2.1. Breast CAD Algorithm Based on CNN

There has been a surge in CNN-based breast CAD algorithms in recent years. A series of detection algorithms initially designed for natural images have been adapted for breast lesion detection [10,11], achieving performance levels that approach or even surpass human interpretation. Building upon this foundation, further research has refined classic frameworks widely used in natural image tasks, tailoring them to specific lesion-detection tasks. For instance, Hossain et al. [12] utilized YOLOv5 for breast lesion detection and proposed augmenting the dataset using false positive samples. Li et al. [13] incorporated breast spatial information extracted using Gabor filters as auxiliary data, concatenating it with the original images and inputting them into Faster-RCNN for structure-distortion detection, thereby enhancing the sensitivity of the detection model. This underscores the effectiveness of incorporating prior breast information in breast lesion-detection tasks. Wang et al. [14] introduced the Context Attention Pyramid Network (CAPNet), selectively fusing feature maps between adjacent layers by computing context feature-selection fusion matrices. Ke et al. [15] proposed the Adaptive Scale Module (ASM) and Feature Refinement Module (FRM) based on U-Net, learning high- and low-dimensional scale features and enhancing channel dimension features. Xu et al. [16] presented the Adaptive Receptive Field Network (ARF-Net), which uses convolutional kernels of different sizes to capture multi-scale information and then merges the acquired two-scale information.

However, the feature maps in the FPN of these algorithms present several drawbacks:

(1) When predicting target positions using high-level feature maps, the algorithms fail to exploit the detailed features from lower layers. High-level feature maps primarily contain semantic information about lesions, yet the prediction results derived from these maps lack the assistance of detailed features from lower layers. Consequently, detection algorithms based on FPN may struggle to precisely localize target edges.

(2) The top-down and layer-by-layer feature-fusion mechanism of FPN primarily emphasizes feature interactions between adjacent layers, causing a gradual attenuation of high-level features as they propagate to lower layers. As a result, lower-level feature maps fail to acquire effective semantic information from higher layers to guide their detection process [17].

(3) Classic FPN assigns equal attention weights to all pixels during the feature-fusion stage, resulting in dense glandular regions in the background receiving the same importance as other tissues. This lack of discrimination makes it challenging to distinguish between lesion masses and dense glandular regions.

Considering these limitations, breast mass-detection algorithms based on FPN may not achieve the desired performance improvements. Therefore, we propose the CA-FPN to model global correlations of multi-scale feature maps, enhancing the effectiveness of cross-scale feature interactions and the directness of feature fusion. Additionally, we propose weighting intermediate feature maps based on breast density information to enhance the network’s focus on dense breast regions.

### 2.2. Boundary Modeling in Medical Image Detection

Lesion detection in medical images is a challenging task due to the complexity of the scenes. In DBT images, the similarity between tumor lesions and normal tissue backgrounds often results in unclear tumor boundaries. The issue of uncertain object boundaries is also prevalent in natural images, and in recent years, scholars have proposed various solutions to address this problem. In 2019, He et al. [18] introduced the concept of modeling boundary box probability distributions for object detection in natural images. They observed that boundary box labels often exhibit ambiguity due to inaccuracies in bounding box annotations, partial occlusion of the detected object, or blurred object boundaries. Traditional object-detection frameworks predominantly utilize L-norm losses (e.g., L1 loss, L2 loss, SmoothL1 loss [19]) or IoU-based losses (IoU loss [20], GIoU loss [21], DIoU loss [22]) for bounding box regression tasks. These loss functions measure the discrepancy between predicted bounding boxes and label boxes, guiding parameter updates in the backpropagation algorithm, but fail to reflect the model’s evaluation of label uncertainty. He et al. assumed that the predicted bounding box coordinates follow a Gaussian distribution and modeled the probability distribution of bounding boxes by predicting the mean and standard deviation of the Gaussian distribution. The model’s output mean represents the predicted bounding box coordinates, while the variance indicates the model’s uncertainty assessment of the prediction. Similarly, Choi et al. [23] utilized Gaussian distribution modeling for predicted boxes, modifying the multiscale outputs of the classical detection model YOLOv3 [24] to predict the mean and standard deviation of Gaussian distributions.

Given the complexity and diversity of tumor boundary issues in DBT compared to natural images, modeling uncertain boundaries based on the Dirac or Gaussian distribution is insufficiently flexible and poses challenges when directly applied to mass-detection problems. Inspired by ref. [25], we propose a DBT mass-detection bounding box modeling method based on arbitrary probability distribution models. Specifically, instead of predicting a single position coordinate, we predict a set of data representing the probability distribution function of discretized predicted coordinates. This approach allows for more flexible modeling of bounding box probability distributions.

## 3. Methodology

### 3.1. Overall Architecture

**Figure 2:**
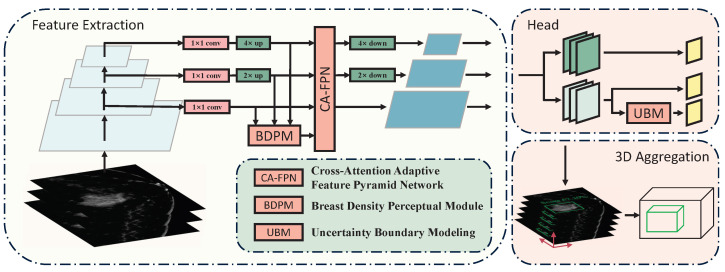
Overall architecture of proposed method. The BDPM and CAFPN are used to further integrate and collect the features extracted by the backbone. The UBM is placed in the regression branch of the detection head to predict more accurate 2D bounding boxes.

The overall architecture of our proposed method is illustrated in Figure 2. After being unified to the same size, the last three feature maps of different scales from the backbone are fed into the CA-FPN for cross-scale feature interaction. Simultaneously, the BDPM module utilizes information on breast density distribution to weight the intermediate feature maps. The feature maps are then restored to their original sizes for downstream tasks. The UBM module learns the discretized probability distribution of bounding box positions and obtains two-dimensional bounding box position predictions through weighted summation.

### 3.2. Cross-Attention Adaptive Feature Pyramid Network

The classical FPN network adopts a top-down feature-fusion approach, which prevents bottom-level features from propagating to higher-level features. However, these bottom-level features often contain more structural and detailed information that is beneficial for breast lesion detection. Moreover, FPN networks only focus on feature fusion between adjacent layers, leading to a gradual decrease in semantic information as it propagates from higher to lower layers, thus hindering effective guidance of bottom-level features by higher-level semantics.

**Figure 3:**
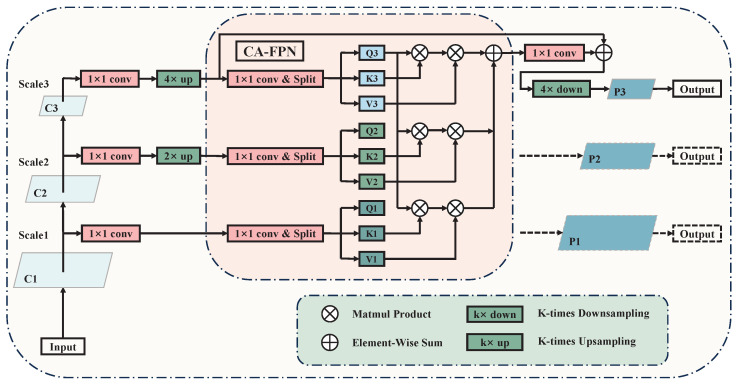
Architecture of CA-FPN. The module directly connects deep features with shallow features, preventing the gradual attenuation of feature transmission seen in traditional FPN.

An intuitive solution is to concatenate feature maps of different levels after sampling them to a unified size along the channel dimension. However, such rough fusion lacks feature selection, resulting in the mixing of noise from different dimensions during feature fusion. Furthermore, it fails to address the semantic gap between feature maps at different levels. To solve these problems, we designed the Cross-Attention Adaptive Feature Pyramid Network (CA-FPN) to model the global correlation of multi-scale feature maps, which improves the effectiveness of cross-scale feature interaction and the directness of feature fusion. The details of the CA-FPN are shown in Figure 3.

Denoting the $i^{th}$ scale feature map as $F_{i}\in R^{C\times H\times W}$, the corresponding output feature map $O_{i}\in R^{C\times H\times W}$ can be computed using the following formula:

(1) $$O_{i}=W_{i}\left(\sum_{j=1}^{N}CoAtt\left(F_{i},F_{j}\right)\right)+F_{i}$$

where *i* and *j* are indices representing the scale of the feature maps, N denotes the total number of feature maps at different scales, and CoAtt(·) represents the cross-attention computation function. From the equation, it can be observed that CA-FPN computes the cross-attention between the feature maps of the current scale and any other scale, including self-attention within the feature maps of the current scale. Subsequently, pixel-wise addition is performed to achieve weight fusion, ensuring the directness and effectiveness of the interaction between feature maps at different scales.

The design of CoAtt(·) is inspired by the self-attention layer in the Transformer [26]. It computes the global correlations between feature maps through matrix multiplication, which can be expressed by the following formula:

(2) $$CoAtt=sofmax\left(Q_{i}{\left(F_{i}\right)}^{T}\times K_{j}\left(F_{j}\right)\right)\times V_{j}\left(F_{j}\right)$$

where *i*, *j*, and *k* represent the scale indices of the feature maps; softmax(·) denotes the normalization function; Q(·), K(·) and V(·) represent the feature mapping functions, with weights not shared among different scales, used to map the input feature maps into a different dimensional space. In this experiment, 1 × 1 convolutions are employed to map input feature maps of dimension C to feature spaces of dimension C/2, aiming to reduce computational complexity and memory usage. The superscript T denotes matrix transpose.

The feature fusion in CA-FPN is bidirectional, meaning that the prediction branch of the top-level feature map can benefit from the guidance of detailed features from the bottom level, thereby enhancing the accuracy of boundary localization. Additionally, in CA-FPN, feature maps from different scales can be fused directly, avoiding the issue of gradual attenuation of high-level features as they pass down to lower levels, which is a common problem in traditional FPNs.

### 3.3. Breast Density Perceptual Module

**Figure 4:**
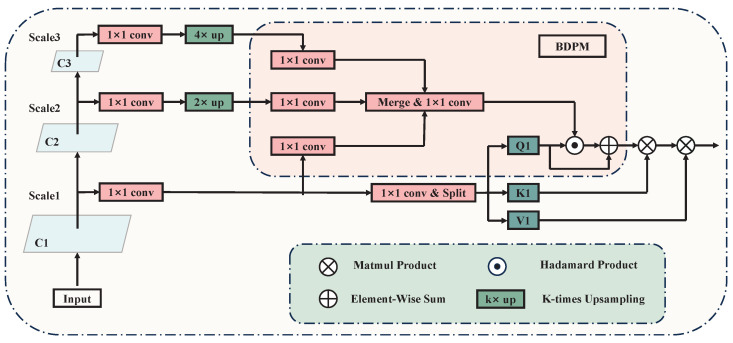
Architecture of BDPM. The module weights the intermediate features of the network using breast density information to enhance the network’s focus on dense breast regions.

Considering variances in glandular tissue distribution and local density, certain densely packed glandular tissues exhibit features similar to tumor lesions, leading to potential misdiagnoses. Traditional CNN algorithms typically assign uniform attention weights to all pixels across the entire image during the feature-fusion stage. Consequently, densely packed glandular regions within the background are afforded equal importance as other tissues in the feature maps forwarded to the classifier. This makes it difficult to effectively discern tumor lesions from dense glandular regions, resulting in some densely packed glandular tissues being erroneously identified as false positive samples. Inspired by Li et al. [13] to address this issue, we propose the Breast Density Perceptual Module (BDPM), which weights the intermediate features of the network using breast density information to enhance the network’s focus on dense breast regions. The details of the BDPM module are shown in Figure 4.

Denoting the $i^{th}$ scale feature map as $F_{i}\in R^{C\times H\times W}$ and the feature map to be weighted as $Q_{i}\in R^{C\times H\times W}$, the corresponding output feature map $R_{i}\in R^{C\times H\times W}$ can be computed using the following formula:

(3) $$R_{i}=W_{i}\left(D\left(F_{1},F_{2},...,F_{n}\right)\right)\circ Q_{i}+Q_{i}$$

where *i* represents the scale indices of the feature maps, *n* represents the total number of feature maps for different scales, D(·) represents the breast density perception function, and ∘ represents the Hadamard product. During the training process, the perception of breast density is supervised by manually extracted breast density maps denoted as $D_{k}$, which are obtained by processing the corresponding slices of DBT images. Specifically, the original slice $I_{k}$ undergoes Gamma filtering initially, which suppresses low-intensity regions while preserving high-intensity areas, thereby retaining information about high-density breast regions. Subsequently, the resultant image undergoes Gaussian filtering to smooth breast areas and remove fine texture details. Finally, normalization is applied, yielding the breast density supervisory map $D_{k}$. The corresponding computational formulation is formulated as below:

(4) $$D_{k}=N\left(G\left(\Gamma \left(I_{k},\gamma \right)\right)\right)$$

where N(·) represents the normalization function, G(·) represents the Gaussian smoothing filter, Γ(·) represents the Gamma correction function, and γ represents the parameter of the gamma filter, used to control the expansion effect of the function on different gray scales. In experiments, the value of γ was set to 0.6.

The BDPM aims to identify regions containing relatively efficient features within the feature map and then focus more on these regions when fusing features from different layers. It is worth noting that by learning the density maps directly from the feature maps and optimizing them through both the module-specific and main network loss functions, the BDPM also mitigates the negative impact of noise present in manually extracted breast density maps on the network. This ultimately improves the accuracy of feature fusion and the overall detection performance.

### 3.4. Uncertainty Boundary Modeling

The $L_{n}$ norm loss function used in classical object-detection frameworks can be regarded as directly predicting the Dirac distribution of coordinates, lacking an estimation of uncertainty regarding the bounding boxes. Furthermore, the density of lesions resembles glandular tissue in DBT, leading to an uncertainty boundary due to various factors. And consequently, the distribution of label boundaries becomes complex, rendering the use of fixed distribution functions inflexible. Inspired by reference [25], we propose the use of a function R(x) that follows an arbitrary distribution to fit the distribution of breast lesion labels. Considering the object-detection tasks in real-world scenarios, the predicted results of bounding box coordinates often fall within a certain range (e.g. the image boundaries). Therefore, assuming the labels follow distribution $y\in [y_{1},y_{k}]$, the integral form of the predicted results $\hat{y}$ can be equivalently expressed as:

(5) $$\hat{y}=\int_{y_{1}}^{y_{k}}R\left(x\right)xdx$$

Since it is challenging for CNNs to directly predict continuous functions that follow arbitrary distributions, the distribution function R(·) is discretized into $R^{\prime }\left(\cdot \right)$, and the integral interval $\left[y_{1},y_{k}\right]$ is uniformly discretized into $\left[y_{1},y_{1},...,y_{k-1},y_{k}\right]$. Consequently, the corresponding predicted result $\sum_{i=1}^{k}R^{\prime }\left(y_{i}\right)=1$ can be expressed as:

(6) $$\hat{y}=\sum_{i=1}^{k}R^{\prime }\left(y_{i}\right)y_{i}$$

In the experiments, we predicted a vector of length *k* to represent the discrete distribution function $R^{\prime }\left(\cdot \right)$. However, due to the independence of feature extraction between convolutional kernels, each element of the predicted discrete distribution vector is independently computed by different convolutional kernels, which makes it challenging to effectively model the correlations between elements of the distribution vector. Therefore, we introduce a learnable distribution-correction unit, which adaptively obtains more accurate discrete distribution modeling results for the bounding box. The final predicted result $\hat{y}$ can be expressed as:

(7) $$\hat{y}=\sum_{i=1}^{k}W_{i}R^{\prime }\left(y_{i}\right)y_{i}$$

where $W_{i}$ represents the weight coefficients of the distribution modeling unit. The implementation employs grouped convolution with 1 × 1 convolutional kernels, where the number of groups was set to four to ensure the independence of the distribution modeling for the four boundary positions. To enhance the stability of the model during the early stages of training, the distribution-correction unit incorporates prior information about the probability distribution of the labeled boxes through weight initialization. In the experiments, this module is initialized with a Gaussian distribution. It is important to note that this procedure provides only a weak prior for the boundary box position distribution modeling and does not compromise the flexibility of the final algorithm in predicting probability distributions.

### 3.5. Loss Function

The over loss function $L_{\mathrm{total}}$ of the model includes five components: classification loss $L_{\mathrm{cls}}$, centerness loss $L_{\mathrm{cen}}$, uncertainty boundary modeling loss $L_{\mathrm{mod}}$, bounding box regression loss $L_{\mathrm{reg}}$, and density map generation loss $L_{\mathrm{dm}}$. In this study, the cross-entropy loss is selected as $L_{\mathrm{mod}}$, and the Smooth L1 loss is used for $L_{\mathrm{dm}}$. The other loss functions are consistent with the settings in FCOS [27]. The overall loss is as follows:

(8) $$\begin{array}{c} \begin{array}{cc} L_{total} & =\frac{\lambda_{cls}}{N_{poz}}\sum_{x,y}L_{clz}\left(p_{x,y},c_{x,y}^{*}\right)\\ & \;+\frac{\lambda_{cen}}{N_{poz}}\sum_{x,y}I_{c_{x,y}^{*}>0}L_{cen}\left(t_{x,y},t_{x,y}^{*}\right)\\ & \;+\frac{\lambda_{mod}}{N_{poc}}\sum_{x,y}I_{c^{*}x,y>0}L_{mod}\left(m_{x,y},m_{x,y}^{*}\right)\\ & \;+\frac{\lambda_{reg}}{N_{poc}}\sum_{x,y}I_{c^{*}x,y>0}L_{reg}\left(n_{x,y},n_{x,y}^{*}\right)\\ & \;+\frac{\lambda_{dm}}{N_{poz}}\sum_{x,y}L_{dm}\left(d_{x,y},d_{x,y}^{*}\right) \end{array} \end{array}$$

where $N_{poz}$ represents the number of positive samples; *x* and *y* represent the position coordinates on feature maps of all scales; $\left(p_{x,y},c_{x,y}^{*}\right)$, $\left(t_{x,y},t_{x,y}^{*}\right)$, $\left(m_{x,y},m_{x,y}^{*}\right)$, $\left(n_{x,y},n_{x,y}^{*}\right)$, $\left(d_{x,y},d_{x,y}^{*}\right)$ denote the predicted results and labels for different tasks; $I_{c_{x,y}^{*}>0}$ is a positive sample labeling function, meaning that the model only computes $L_{qlt}$, $L_{mod}$ and $L_{reg}$ for foreground pixels. λ serve as the coefficients for each component of the loss function, typically set to a value between 0 and 1 to balance the convergence rates of the different components during training. In this study, we assigned the values 1, 1, 0.25, 1, and 0.1 to $\lambda_{cls}$, $\lambda_{cen}$, $\lambda_{mod}$, $\lambda_{mod}$ and $\lambda_{dm}$, respectively.

### 3.6. 3D Aggregation

**Figure 5:**
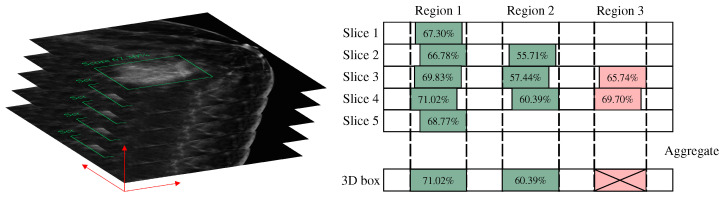
Three-dimensional aggregation. These 2D detection results are fused along the z-axis to yield the final 3D detection results.

In this study, the algorithm employs a slice-by-slice detection and fusion approach to obtain the final three-dimensional prediction box, necessitating a post-processing operation for merging detection boxes. During the testing phase, all slices of each DBT volume are sequentially fed into the model to obtain the corresponding 2D detection boxes. These 2D detection boxes are then fused along the z-axis to yield the final 3D detection results. The specific method for merging 2D detection boxes into a 3D box is shown in Figure 5. First, the Intersection over Union (IoU) of the 2D detection boxes in adjacent slices is calculated. If the IoU exceeds a threshold of 0.5, the detections are considered to be of the same lesion. Furthermore, only if a lesion is detected in at least three consecutive slices will the bounding boxes be merged. For example, if a bounding box is detected in only two slices, as in the case of region 3, it is not considered a valid detection. The final 3D bounding box is the union of the 2D bounding boxes, ensuring that the entire detection is maximally included. The confidence score of the 3D bounding box is taken as the maximum confidence score among the 2D detection results.

## 4. Experiment

### 4.1. Materials and Evaluation Metrics

Two DBT datasets were used in this study, which were, respectively, from the private dataset collected by Suzhou Municipal Hospital and the open source DBT dataset BCS-DBT [28].

The private dataset, collected by Suzhou Municipal Hospital, affiliated with Nanjing Medical University, consists of 310 DBT volumes from 155 patients (approved by the Ethics Committee of Nanjing Medical University; reference number: NMU2022137). All data were acquired using a Hologic Selenia Dimensions 3D Mammography machine. The DBT tomographic images were reconstructed using a filtered back-projection algorithm, with an in-plane pixel spacing of approximately 0.07 mm and a slice thickness of 1 mm. The tube voltage ranged from 27 to 29 kV, and the tube current was 100 mA. Each case included data from both craniocaudal (CC) and medial lateral oblique (MLO) views. A clinician with over five years of experience in DBT clinical diagnosis annotated the lesion locations with 3D bounding boxes, resulting in 310 annotated mass lesions, all of which had pathological confirmation. As shown in Table 1, the dataset was randomly divided at the patient level into training, validation, and test sets. The training and validation sets were divided by 5-fold crossing. To ensure the demographic representativeness of the dataset, we conducted a statistical analysis of the age distribution among the patients, presented in Table 2.

The open source dataset used in this experiment included 309 DBT volumes from 158 patients. The image sizes in the dataset were mostly 2457 × 1996 or 2457 × 1980, with the number of slices ranging from 40 to 100. Most patients typically included DBT data from four positions: left CC, left MLO, right CC, and right MLO. However, some cases might have had missing data for certain positions. The dataset used in this experiment contained 332 mass lesions with pathological diagnosis results. Each lesion was annotated with the center slice position, the coordinates of the bounding box center within the slice, and the lesion size. The study retained the original partitioning of the open source dataset, with the specific data division shown in Table 3. It is worth noting that the BCS-DBT dataset also contains lesions without pathological diagnosis results. The dataset creators classify these lesions as negative samples and do not annotate their locations.

For this study, the primary evaluation metric for detection performance was the mean detection sensitivity under various false positive detection conditions (1, 2, 3, and 4 FPs per DBT). The Free Receiver Operating Characteristic (FROC) and the partial Area Under the FROC Curve (pAUC) [29] were also used to visually show the comprehensive performance of the model.

### 4.2. Implementation Details

Common preprocessing operations were performed on the data, including window width and level adjustment, breast region segmentation based on thresholding, image mean normalization, and scaling the processed images to a resolution of 1000 × 500 to reduce the computational load of the algorithm. For feature extraction, we selected ResNet-50 [30], a network commonly used in medical image analysis tasks [31]. Considering the distribution of lesion bounding box sizes and the downsampling factor of the detection model for the input images, the feature vector length of the UBM was set to 8.

To maintain the spatial positional correlation of slices along the z-axis, we used a three-channel 2D image composed of three adjacent slices as the model input. During training, based on the coordinates of the bounding boxes, the central slice and the two adjacent slices were used as input data.. Additionally, online data-augmentation methods based on random vertical and horizontal flipping were applied to the training data. In the testing phase, all slices were sequentially input into the model along with their adjacent slices, and the 2D detection results were merged into 3D to evaluate the model’s detection performance.

All experiments were conducted on a workstation equipped with a GeForce RTX 3090 GPU. The proposed model was implemented using the PyTorch framework [32] on the MMDetection platform [33] and optimized using the momentum stochastic gradient descent (SGD) algorithm, with the momentum parameter set to 0.9 and the weight decay set to 0.0001. The model weights were initialized with parameters pre-trained on the ImageNet dataset [34]. The batch size for each iteration was set to 4. To prevent overfitting, an early stopping strategy was adopted, with the maximum number of training epochs set to 45. The learning rate was set to 0.001 during the initial training phase and adjusted to 0.0001 at the 30th epoch.

### 4.3. Results

Considering the primary improvements of the proposed algorithm in the FPN structure and regression method, we compared it against a series of mainstream object-detection algorithms that incorporate the FPN structure, including Faster R-CNN [35], Cascade R-CNN [36], RetinaNet [37], GFL [25], and FCOS [27]. Additionally, to validate the performance of our algorithm, we compared it with several state-of-the-art object-detection algorithms, specifically YOLOv9 [38] and Co-DINO [39]. To ensure a fair comparison, all networks were trained with the same input data and parameter settings as our proposed algorithm, except for Co-DINO, which has a slower convergence and thus had its maximum training epoch set to 70. All models used ResNet-50 pre-trained on the ImageNet dataset for the backbone feature-extraction network, and the final prediction results underwent the same merging operation to obtain the 3D prediction boxes.

**Figure 6:**
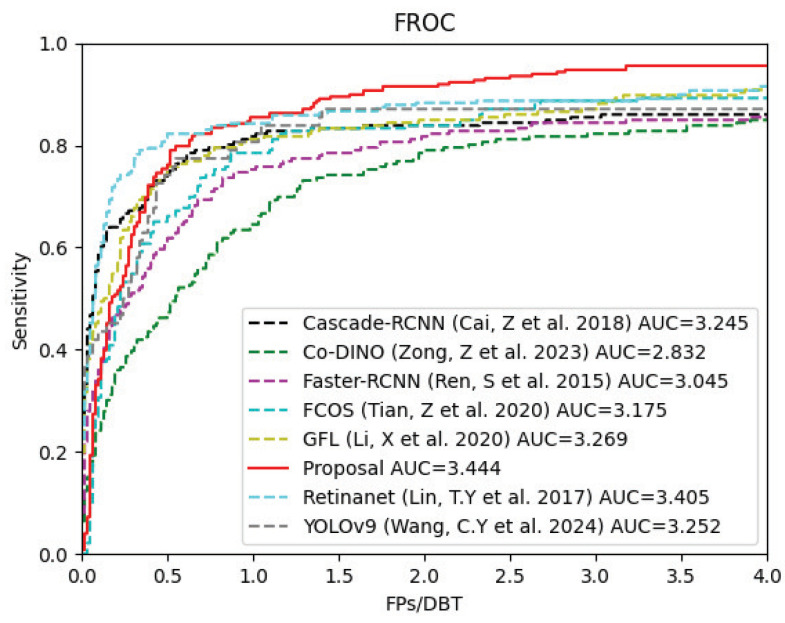
The FROC curves of the comparison methods [25,27,35,36,37,38,39] on mass-detection task.

The experimental results on the private dataset, presented in Table 4, indicate that our network outperforms the other methods across most evaluation metrics. The FROC curves for each algorithm, shown in Figure 6, provide a clear visualization of their performance. Our algorithm achieved the optimal pAUC, demonstrating superior performance. Moreover, in terms of the sensitivity when the FPs/DBT ratio is 1, although our method does not take the leading position, it only lags behind the first-place method by 0.65%. This discrepancy may arise because the BDPM module inadvertently assigns higher weights to certain high-density false positive samples, resulting in higher detection scores. Nonetheless, overall, the proposed method demonstrates an effective ability to differentiate the majority of lesions from false positive samples.

Faster R-CNN [35] and Cascade R-CNN [36] are both two-stage object-detection algorithms based on CNNs. During the training phase, Faster R-CNN relies on fixed IoU thresholds to distinguish between positive and negative samples, which leads to insensitivity in detecting small lesions. Cascade R-CNN employs a sequential multi-stage prediction strategy that incrementally increases the IoU threshold, thereby achieving higher precision in bounding box regression. This approach yields better performance than Faster R-CNN. However, the detection performance still falls short of expectations.

Co-DINO [39] is an object-detection algorithm based on the Transformer architecture, utilizing self-attention mechanisms and feed-forward neural networks to encode features from extracted feature maps. Compared to CNNs, Transformers can better model global relationships in the image. However, these algorithms are less sensitive to image details, which can be detrimental in tasks like breast mass detection where distinguishing features between breast masses and dense glandular tissue may be overlooked. As a result, the performance of Co-DINO falls significantly short of the anticipated benchmarks. To address this issue, our CA-FPN design incorporates pathways for direct interaction between deep and shallow features, allowing deep features to be informed by detailed information from shallow features. Additionally, we developed the BDPM module to enhance the focus on high-density breast regions during feature fusion, guiding the network to better differentiate between breast masses and dense glandular tissue.

RetinaNet [37], FCOS [27], and YOLOv9 [38] are representative single-stage object-detection algorithms based on CNNs. RetinaNet and FCOS both employ the FPN structure for feature enhancement, where features are passed from deeper to shallower layers. However, this top-down feature fusion does not allow deep features to be guided by detailed information from shallow features. Moreover, the stepwise feature fusion results in a gradual loss of deep semantic information in shallow features. Consequently, the FPN’s feature fusion is insufficient, as deep features miss out on detailed image information crucial for mass differentiation, and shallow features lack comprehensive semantic guidance. YOLOv9 introduced the Programmable Gradient Information (PIG) framework, which aggregates gradient information of target objects from different layers of the deeply supervised FPN, achieving excellent detection performance in our task. Although YOLOv9 incorporates a bottom-up feature-fusion process, the interaction between shallow and deep features remains indirect, and the issue of information attenuation persists. Therefore, we propose CA-FPN to achieve direct global correlation modeling between multi-scale feature maps, enhancing the directness and effectiveness of cross-scale feature interactions and avoiding the information-attenuation problem associated with stepwise feature transmission.

GFL [25] pioneered the use of discrete distribution modeling in natural image detection tasks and demonstrated effective detection performance in our experiments on DBT breast mass detection. Nonetheless, GFL’s modeling approach can be further optimized for mass-detection tasks. GFL predicts discrete boundary distribution functions directly through convolutional layers, where each element of the predicted discrete distribution vector is independently calculated by different convolutional kernels. This independence limits the effective modeling of correlations between elements in the distribution vector. Breast mass boundaries, compared to those in natural images, are often more ambiguous and require such internal correlations to aid in boundary regression. Thus, we introduce a discrete distribution-correction module composed of grouped convolutions with 1 × 1 kernels, which adaptively yields more accurate discrete boundary distribution-modeling results. Additionally, this module can incorporate prior information on the probability distribution of label boxes through weight initialization, enhancing model stability during the early stages of training.

To further validate the effectiveness of our proposed method, we compared our proposed method with state-of-the-art models on the BCS-DBT dataset. As presented in Table 5, our network outperforms the other methods across all evaluation metrics. It is worth mentioning that, Co-DINO performs poorly on public datasets, likely due to the more complex data characteristics in this dataset, which contain more hard samples. As a result, transformer-based algorithms require more data and training iterations to effectively learn from such challenging examples.

### 4.4. Detection Result Visualization of Different Models

**Figure 7:**
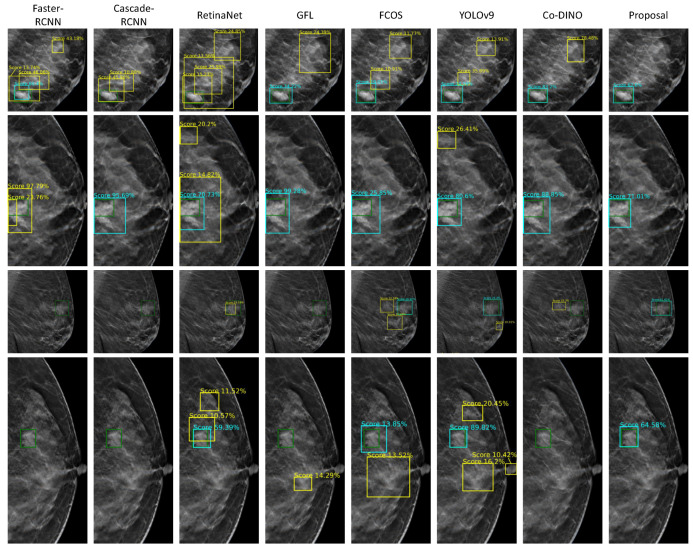
The detection result visualization of different models, in which the green boxes represent ground truth, the aqua blue boxes represent true positive results, and the yellow boxes represent false positive results.

We further visualized the detection results of different models at the 2D slice level. As shown in Figure 7, the ground truth and the true positive results are depicted by green and aqua blue boxes, respectively, while some false positive results are highlighted in yellow boxes.

The previously mentioned algorithms exhibited varying degrees of false positives in dense glandular regions. This issue likely arises because the similar appearance of dense glandular tissue and masses increases the difficulty of differentiation. These algorithms fail to effectively integrate image feature information from different levels that is crucial for distinguishing such regions with their indirect and insufficient cross-layer feature-fusion methods. Notably, the proposed method successfully identifies these regions that are easily mistaken for FPs. This performance improvement may benefit from the CA-FPN, which enables effective integration of high-level semantic features and low-level detailed features. Additionally, the BDPM enhances the network’s ability to differentiate between high-density breast regions and masses. The UBM further adapts to the complex and variable boundaries, contributing to better performance in distinguishing these challenging cases.

### 4.5. Heatmap Visualization of the Detection Results Using Grad-CAM

To further illustrate the interpretability of the proposed method, we employed the Grad-CAM (Gradient-weighted Class Activation Mapping) technique to visualize the intermediate feature maps derived from the detection results. Grad-CAM is a robust method for visualizing class-specific activation regions within Convolutional Neural Networks (CNNs). This approach provides valuable insights into the decision-making process of neural networks during classification tasks by identifying and emphasizing the image regions that significantly contribute to the final predictions.

**Figure 8:**
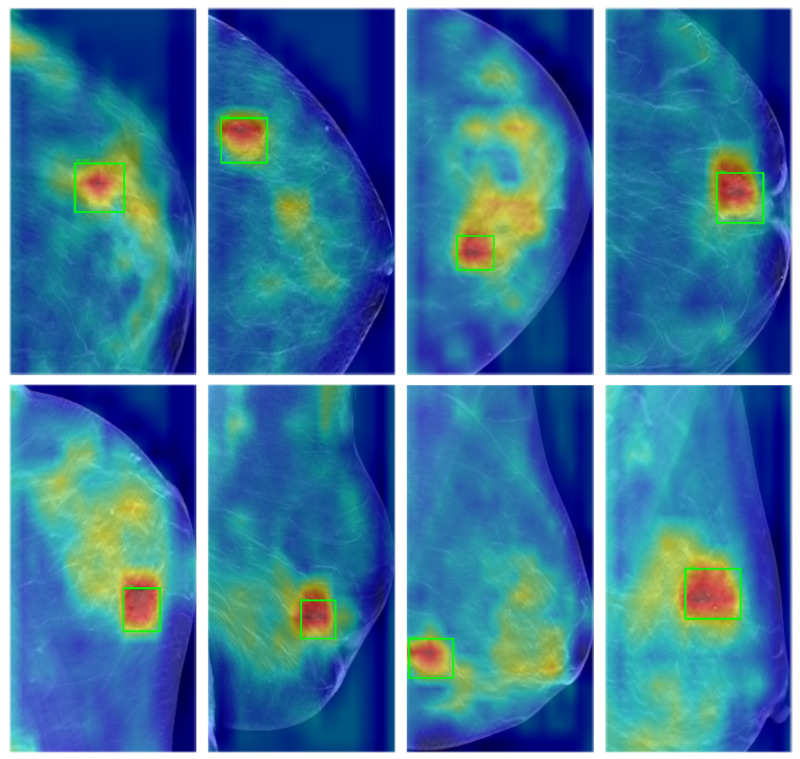
The heatmap visualization of the detection results using Grad-CAM, in which regions highlighted in red indicate areas of the feature map that receive higher attention from the network and regions depicted in blue represent areas with lower attention from the network. The green boxes represent ground truth. The proposed method effectively focuses on the ground truth regions, thereby achieving accurate detection results.

As shown in Figure 8, regions highlighted in red indicate areas of the feature map that receive higher attention from the network, whereas regions depicted in blue represent areas with lower attention from the network. For a more intuitive visualization of the detection performance, the ground truth regions are delineated with green bounding boxes. As illustrated, The proposed method demonstrates a strong ability to focus on the ground truth areas, consequently producing accurate and reliable detection results.

### 4.6. Ablation Study

We further conducted ablation experiments on the proposed method to validate the effectiveness of its components. The data split and evaluation metrics in the ablation experiments were consistent with those of the private dataset in the comparative experiments.

#### 4.6.1. The Effectiveness of the Algorithm Module

We performed ablations on each of the proposed algorithm modules, and the results are presented in Table 6. The baseline for these experiments is the standard FCOS network. Initially, we added the CA-FPN module to the baseline, resulting in improved detection sensitivity compared to the baseline, which indicates that the CA-FPN, through efficient cross-scale feature fusion, increased the model’s lesion-detection rate. Building on this, we integrated the BDPM module, which further enhanced the detection sensitivity. This improvement demonstrates that by weighting breast density distribution, the BDPM enhances the network’s focus on dense breast regions, thereby improving the detection of breast masses. Finally, we integrated the UBM and conducted further experiments. The results showed that the UBM significantly achieved better sensitivity at all average false positive rates, proving the effectiveness of uncertainty modeling for blurred boundaries in DBT mass-detection tasks.

#### 4.6.2. The Effectiveness of the UBM Branch

To evaluate the effectiveness of our proposed UBM branch for modeling blurred boundaries, we conducted experiments based on the standard GFL network. The results are shown in Table 7, where “+UBM” indicates the results of replacing the original regression branch of the GFL model with the UBM branch. The improved model achieved better detection sensitivity. Additionally, the final average detection sensitivity was superior to those of the GFL model, demonstrating that our improvements in modeling uncertain boundaries contribute to enhancing the overall lesion-detection performance of the model.

#### 4.6.3. The Selection of k Value for the UBM Branch

We conducted experiments to determine the optimal k value for the UBM branch, where k represents the length of the predicted boundary discrete distribution vector. The experiments’ results are shown in the Table 8. As indicated by the results, the algorithm achieved optimal detection performance when the k value was set to 8. When the k value was set to 4, the predicted boundary distribution range was too small, failing to cover the boundary range of larger masses, resulting in poorer model performance. Conversely, when the k value was set to 16 or 32, the predicted boundary distribution range far exceeded the boundary range of the mass, introducing excessive irrelevant information that interfered with the prediction of the discrete boundary distribution. This interference led to poorer lesion-detection performance, with the model’s effectiveness deteriorating further as the k value continued to increase.

## 5. Discussion

For the fusion of features from different levels, we initially attempted to simply concatenate feature maps from various levels along the channel dimension after resampling. However, the detection results for breast masses were not as expected. We hypothesize that this is partly because the detection targets in DBT images are more ambiguous compared to natural images, and the background noise is more complex. Indiscriminate feature fusion limits the enhancement effect on the target features, and also mixes and amplifies the noise extracted from different levels. Additionally, there is a semantic gap between feature maps from different levels, and simple channel concatenation cannot achieve the desired feature-fusion effect. Inspired by cross-attention mechanisms, we designed the CA-FPN network. By calculating cross-attention parameters between features at different levels, we selectively enhanced the features to be fused, ensuring the effectiveness of feature interactions across different levels. This approach also helps to mitigate the semantic gap between feature layers to some extent.

However, despite the improvements over existing methods, our proposed method still has limitations. Firstly, while the BDPM enhances the network’s ability to differentiate between mass lesions and dense glandular regions by weighting the feature maps, it also inadvertently increases the classification scores of false positives in dense glandular areas in the background. In most cases, this issue can be mitigated by setting appropriate thresholds; however, it remains ineffective for challenging samples where masses are heavily obscured by dense glandular tissue. Addressing high-confidence false positives arising from challenging samples with certain dense glandular tissue remains a critical issue to resolve in future work. Secondly, the current algorithm’s approach of concatenating adjacent slices into a three-channel image input only captures information between neighboring slices. Since most masses span multiple layers of slices, this method limits the ability to extract comprehensive three-dimensional information about the lesions.

Future work will focus on designing targeted strategies for distinguishing these challenging samples and developing a more comprehensive 3D feature extraction module. To address these high-confidence false positives, one potential approach is to implement the strategy of Hard Example Mining for targeted training. Another viable solution involves utilizing generative networks, such as diffusion models, to synthesize additional challenging samples that mimic these difficult cases. Incorporating these augmented datasets into the training process could significantly improve the model’s ability to differentiate and accurately classify such complex instances. These will aim to achieve superior detection performance for DBT breast masses by effectively capturing the extensive three-dimensional information of the lesions.

## 6. Conclusions

In this study, we present a novel and efficient network designed to address the challenges of mass detection in DBT. Our proposed CA-FPN significantly advances the classic FPN by integrating feature maps through a cross-attention mechanism, enabling efficient and direct cross-scale feature interactions. Furthermore, the BDPM dynamically incorporates breast density information to weight intermediate feature maps, effectively guiding the network to prioritize dense breast regions. For boundary regression, the UBM framework simultaneously models the distribution function of predicted box positions and quantifies the uncertainty in the predicted coordinates, leading to more precise and reliable bounding box predictions. Extensive experimental results on two benchmark datasets demonstrate the superior performance of our proposed method over existing approaches. Despite these advancements, our current method has certain limitations. For instance, it faces challenges in detecting masses heavily obscured by dense glandular tissue and lacks the capability to fully exploit multi-layer inter-slice three-dimensional information.

In future work, we will focus on designing more targeted strategies for distinguishing these hard samples and developing a more comprehensive 3D feature-extraction module. By integrating these enhancements into our current framework, we aim to further improve the accuracy and robustness of breast mass detection in DBT, ultimately contributing to more reliable early diagnosis and clinical decision-making.

## Figures and Tables

**Table 1:** The five-fold cross-validation partitioning of the private dataset.

	Cases	Volumes	Focuses
1st fold	25	50	50
2nd fold	25	50	50
3rd fold	25	50	50
4th fold	25	50	50
5th fold	24	48	48
test set	31	62	62

**Table 2:** The age distribution among the patients in the private dataset.

Age	Percentage
20–30	0.55
30–40	8.20
40–50	15.85
50–60	28.14
60–70	27.32
70–80	17.21
80–90	2.73

**Table 3:** The partitioning of the BSC-DBT dataset.

	Cases	Volumes	Focuses
train set	68	132	147
validation set	36	68	68
test set	54	109	121

**Table 4:** The result of comparison experiments of the MC detection task on the private dataset. ‘STD’ refers to the standard deviation of the average sensitivity results from five experiments in a five-fold cross-validation. The best experimental results under the current conditions are highlighted in bold. (FPs/DBT: false positives per DBT volume.)

Methods	Sensitivity (%)					
	**FPs/DBT = 1**	**FPs/DBT = 2**	**FPs/DBT = 3**	**FPs/DBT = 4**	**Average**	**STD**
**Faster-RCNN [35]**	73.87	82.26	84.51	85.81	81.61	**0.009**
**Cascade-RCNN [36]**	82.58	84.51	86.77	87.10	85.24	0.017
**RetinaNet [37]**	**85.48**	88.71	90.02	91.62	88.95	0.012
**GFL [25]**	80.32	85.48	88.71	91.29	86.45	0.031
**FCOS [27]**	73.23	80.97	85.48	86.78	81.61	0.072
**YOLOv9 [38]**	76.62	80.65	80.65	80.65	79.64	0.058
**Co-DINO [39]**	65.81	80.00	82.90	85.16	78.47	0.012
**Proposed**	84.83	**89.68**	**94.19**	**95.48**	**91.05**	0.011

**Table 5:** The result of comparison experiments of the MC detection task on the BCS-DBT dataset. The best experimental results under the current conditions are highlighted in bold. (FPs/DBT: false positives per DBT volume.)

Methods	Sensitivity (%)				
	**FPs/DBT = 1**	**FPs/DBT = 2**	**FPs/DBT = 3**	**FPs/DBT = 4**	**Average**
**Faster-RCNN [35]**	29.75	45.98	49.59	57.02	44.83
**Cascade-RCNN [36]**	48.76	54.55	61.98	67.77	58.26
**RetinaNet [37]**	32.23	46.28	57.85	62.81	49.79
**GFL [25]**	41.32	52.07	57.02	62.81	53.30
**FCOS [27]**	48.76	67.77	74.38	77.69	67.14
**YOLOv9 [38]**	54.29	66.71	74.26	76.70	67.99
**Co-DINO [39]**	28.10	38.84	44.63	47.93	39.87
**Proposed**	**61.16**	**72.73**	**80.17**	**81.82**	**73.96**

**Table 6:** Quantitative evaluation of ablation experiments of the proposed CA-FPN, BDPM, and UBM modules. ’STD’ refers to the standard deviation of the average sensitivity results from five experiments in a five-fold cross-validation. The best experimental results under the current conditions are highlighted in bold. (FPs/DBT: the false positives per DBT volume.)

Methods	CA-FPN	BDPM	UBM	Sensitivity (%)					
				**FPs/DBT = 1**	**FPs/DBT = 2**	**FPs/DBT = 3**	**FPs/DBT = 4**	**Average**	**STD**
**Baseline**				73.22	80.96	85.48	86.77	81.61	0.071
**1**	***√***			79.03	86.45	88.06	89.35	85.72	0.029
**2**	***√***	***√***		78.38	86.45	90.32	91.61	86.70	0.012
**Proposal**	***√***	***√***	***√***	**84.83**	**89.68**	**94.19**	**95.48**	**91.05**	**0.011**

**Table 7:** The result of comparison experiments of the original GFL and the GFL with our UBM. The best experimental results under the current conditions are highlighted in bold. (FPs/DBT: the false positives per DBT volume.)

Methods	Sensitivity (%)				
	**FPs/DBT = 1**	**FPs/DBT = 2**	**FPs/DBT = 3**	**FPs/DBT = 4**	**Average**
**GFL**	80.64	84.84	88.06	90.64	86.04
**+UBM**	**87.09**	**90.00**	**91.29**	**91.61**	**90.00**

**Table 8:** The result of experiments with different k values. The best experimental results under the current conditions are highlighted in bold. (FPs/volume: the false positives per DBT volume.)

k	Sensitivity (%)				
	**FPs/DBT = 1**	**FPs/DBT = 2**	**FPs/DBT = 3**	**FPs/DBT = 4**	**Average**
**4**	70.96	80.32	84.51	88.06	80.96
**8**	**84.83**	**89.68**	**94.19**	**95.48**	**91.05**
**16**	80.00	87.42	91.29	92.26	87.74
**32**	75.80	83.87	89.35	91.93	85.24

## Data Availability

The data that support the findings of this study are available on request from the author, upon reasonable request.
